# Development of semisynthetic saponin immunostimulants

**DOI:** 10.1007/s00044-024-03227-x

**Published:** 2024-05-18

**Authors:** Di Bai, Hyunjung Kim, Pengfei Wang

**Affiliations:** Department of Chemistry, University of Alabama at Birmingham, Birmingham, AL AL35294 USA

**Keywords:** Vaccine adjuvant, Immunostimulant, Saponin, *Momordica cochinchinensis*, VSA-1, VSA-2

## Abstract

Many natural saponins demonstrate immunostimulatory adjuvant activities, but they also have some inherent drawbacks that limit their clinical use. To overcome these limitations, extensive structure-activity-relationship (SAR) studies have been conducted. The SAR studies of QS-21 and related saponins reveal that their respective fatty side chains are crucial for potentiating a strong cellular immune response. Replacing the hydrolytically unstable ester side chain in the C28 oligosaccharide domain with an amide side chain in the same domain or in the C3 branched trisaccharide domain is a viable approach for generating robust semisynthetic saponin immunostimulants. Given the striking resemblance of natural *momordica* saponins (MS) I and II to the deacylated *Quillaja Saponaria* (QS) saponins (e.g., QS-17, QS-18, and QS-21), incorporating an amide side chain into the more sustainable MS, instead of deacylated QS saponins, led to the discovery of MS-derived semisynthetic immunostimulatory adjuvants VSA-1 and VSA-2. This review focuses on the authors’ previous work on SAR studies of QS and MS saponins.

## Introduction

Vaccine adjuvants are increasingly important to vaccine development owing to their ability to enhance and/or modulate antigen-specific immune responses. Currently, only a few immunostimulating organic compounds, in different formulations and combinations, have been approved by the FDA for human use. They are MPL (monophosphoryl lipid A), QS-21 (a saponin isolated from tree bark extract of *Quillaja Saponaria* Molina), and CpG ODNs (oligodeoxynucleotides of cytosine and guanine with phosphodiester backbone) [1–6]. Among them, QS-21 is arguably the most potent immunostimulant, enhancing antigen-specific humoral and cellular immune responses. The combination of QS-21 with MPL in a liposomal formulation, known as AS01b developed by GlaxoSmithKline (GSK), was recently approved for human use in GSK’s shingles vaccine, *Shingrix*®, and malaria vaccine, *Mosquirix*® [7, 8]. Most recently, Novavax’s COVID-19 vaccine, adjuvanted with Matrix-M^TM^ (a QS-21-containing saponin mixture in nanoparticle form) [9–12], was approved by the FDA in 2022. QS-21 in different formulations or combinations with other adjuvant(s) has also shown promise in many candidate vaccines, including those in recent clinical trials [13–16] and preclinical studies [17–46].

However, QS-21 has its own drawbacks. It is a mixture of natural saponins that contains at least two structural isomers: QS-21_xyl_ and QS-21_api_. The only structural difference of these two isomers is that QS-21_xyl_ has a terminal β-D-xylopyranosyl unit at the C28 linear tetrasaccharide while QS-21_api_ has a terminal β-D-apiofuranosyl unit. These saponins are primarily isolated from the tree bark of *Quillaja saponaria* Molina (QS), which is native to Chile. Between 2009 and 2012, harvests of QS biomass increased from 5700 to 11,600 tons, representing an average annual growth of 19.4%. Overexploitation of QS trees has led to severe ecological damage, consequently, strict environmental regulations have been implemented [47]. The sustainable limit of native forests was estimated to be approximately 27,000 tons per year, which would not meet the increasing annual demand [48]. The ongoing efforts to address QS-21 supply shortage include: 1) growing more *Quillaja saponaria* Molina trees in plantations [49], 2) redistributing the consumption of tree bark from food, cosmetic, and veterinary use to QS-21 extraction, 3) extracting QS-21 from the full biomass of the tree [47], 4) extracting QS-21 from leaves of young trees in QS plantations [48], 5) improving isolation yield [50, 51], and 6) producing QS-21 via enzymatic biosynthesis toward a fermenter-based procedure [52, 53]. Despite the promising progress toward addressing the supply shortage of natural QS-21, other issues persist, notably its dose-limiting toxicity and chemical instability in stock solutions, requiring formulation in liposomes or other nanoparticle constructs such as ISCOMs [47, 54–58]. Thus, development of saponin alternatives to QS-21 that retain its desirable adjuvant activity without its drawbacks - such as a wider therapeutic window, simplified formulation, and improved industrial applicability - is highly desirable.

## Approaches to developing QS-21 alternatives

There are two potential approaches to developing alternatives to QS-21: mechanism-based molecular design or structure-activity-relationship (SAR)-based exploration. However, the former is not currently feasible due to limitations in our understanding of the molecular mechanism of QS-21. It remains unclear whether QS-21’s immunostimulating mechanism includes a receptor-dependent component. Prior mechanistic studies indicate that QS-21 does not seem to operate via a depot effect [59]. Synthetic QS-21 analogs showed a potential role in facilitating antigen trafficking by antigen-presenting cells (APCs) from the site of injection to the draining lymph nodes (dLNs) [60]. In the absence of antigen, Matrix-M^TM^ induced a local transient immune response, recruiting and activating central immune cells in dLNs 48 h after subcutaneous injection [61]. Studies show that QS-21 formulated in liposomes targets CD169+ resident macrophages in dLNs [62]. It rapidly accumulates in these macrophages, inducing Caspase-1 activation and subsequent events. For instance, it may recruit innate immune cells, activate dendritic cells (DCs), and presumably trigger MyD88-dependent and antigen-specific cellular and humoral responses. It remains unclear whether a QS-21 receptor is involved in these events, although QS-21 is known not to bind to Toll-like receptors 2 and 4 [63]. It has been postulated that QS-21 might activate DCs in a receptor-independent manner. For instance, it has been proposed that QS-21 could enhance DCs’ uptake of exogenous protein antigens via cholesterol-dependent endocytosis, and then facilitate antigen escape from endosomes to enhance antigen presentation [64, 65]. Additionally, QS-21’s amphiphilic nature can lead to the formation of pores in lysosome membranes, resulting in the release of lysosomal contents and subsequent inflammasome activation and cytokine production. In vitro and in vivo studies have shown that QS-21-containing ISCOMs induce intracellular lipid body (LB) formation specifically in the CD11b^+^ dendritic cell (DC) subset [66]. This phenomenon enhances the cross-presentation of antigens and subsequent T-cell activation [67]. Inflammasomes are frequently targeted by adjuvants due to their ability to stimulate the adaptive immune system [68, 69]. Recent investigations have explored whether and how QS-21 activates NOD-like receptor protein 3 (NLRP3) inflammasomes. It appears that only when paired with MPL, QS-21 can activate NLRP3 inflammasomes, leading to IL-1β or IL-18 production in certain immune cells such as bone marrow-derived dendritic cells (BMDCs) or immortalized mouse macrophages. Furthermore, in a case study, QS-21-adjuvanted HIV-1 gp120 induced significantly higher levels of antigen-specific Th1 and Th2 immune responses in NLRP3-deficient mice compared to wild-type controls [70]. In another instance, QS-21 in a liposomal formulation activated the NLRP3 inflammasome and produced IL-1β in a Caspase-1-dependent manner [62].

**Fig. 1:**
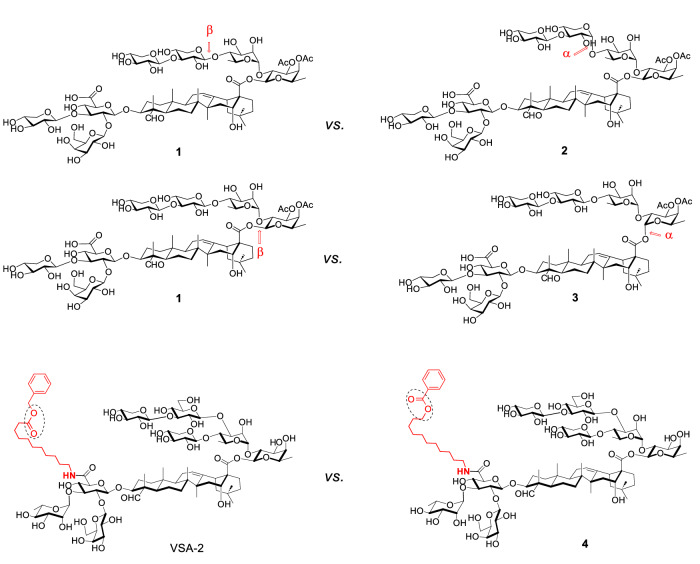
Structure of three pairs of saponin isomers with significantly different adjuvant activities

While the molecular mechanism of QS-21’s adjuvanticity remains elusive, increasing experimental evidence suggests the presence of molecular targets or receptors for QS-21. For example, two saponin isomers with identical hydrophile-lipophile balance (HLB) can exhibit significantly different adjuvant activities. We compared adjuvant activities of two truncated QS-7 analogs, i.e., **1** and **2** (Fig. 1). These diastereomers only differ in the stereochemistry of a glycosidic bond within the C28 linear tetrasaccharide. While analog **1** retained considerable adjuvant activity in potentiating antigen specific IgG1 and IgG2a responses, analog **2** lost its adjuvant activity. A similar trend was observed in the comparison between analogs **1** and **3**, where the only structural difference lies in the C28 glycosidic ester bond, with **1** having a beta glycosidic bond (like QS-7 and QS-21) and **3** having an alpha bond. Additionally, two structural isomers, VSA-2 (VO_0005325; https://vac.niaid.nih.gov/view?id=42) and **4**, which differ only in the terminal functionality of the side chain with a flipped ester group, also demonstrated significantly different adjuvant activities. Studies of these three pairs of isomers suggest that the interaction between a saponin adjuvant and the immune system likely involves a saponin-receptor component; however, the identity of these potential receptors remains unknown.

### Structure-activity-relationship studies of QS saponins

**Fig. 2:**
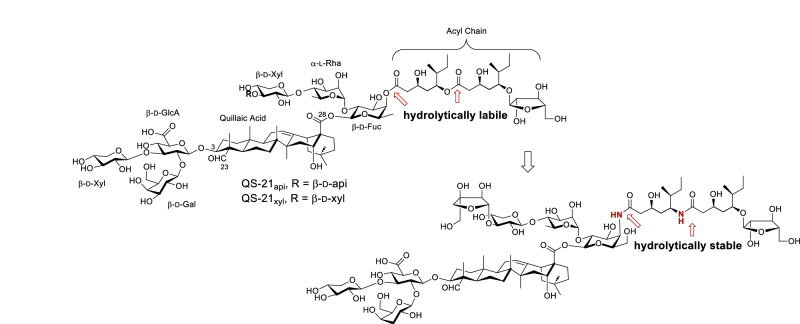
Gin’s SAR studies toward hydrolytically stable QS-21 analogs [73]

Current efforts toward developing QS-21 alternatives primarily focus on SAR studies. QS-21 exhibits hydrolytical instability at physiological pH in stock solutions at ambient temperature. Unlike QS-21, the deacylated byproducts (i.e., QS-21 without the acyl side chain at the C28 fucosyl unit) are incapable of stimulating a Th1 immune response [71, 72]. This chemical instability, along with the dose-limiting toxicity, precludes QS-21 from being used alone as an adjuvant. While proper formulations can improve chemical stability and reduce toxicity, the increased complexity in vaccine formulations is not ideal for clinical evaluation and use. Gin’s laboratory first designed QS-21 analogs equipped with a chemically stable side chain at the original position of the natural side chain [73]. They demonstrated that replacing the indicated ester groups with amide moieties did not reduce the adjuvant activity (Fig. 2). Gin’s laboratory further investigated structurally simplified side chains and found that the array of chiral center within the QS-21 acyl side chain was not essential to maintain adjuvant activity comparable to the natural saponin. Research along this direction, primarily conducted by Gin’s laboratory and Fernandez-Tejada’s laboratory, has been highly productive and extensively reviewed [74–79]. Therefore, this review will not focus on that aspect but instead on our previous work concerning QS saponin SAR studies and the discovery of new VSA adjuvants.

In contrast to Gin’s strategy of developing QS-21 analogs by mimicking the structure of the natural product, i.e., anchoring a simplified side chain at the reducing end of the C28 linear tetrasaccharide, we explored the strategy of incorporating a simple amide side chain at the C3 glucuronic acid site. This strategy was inspired by the success of GPI-0100, a complex mixture of semisynthetic QS derivatives [80, 81]. GPI-0100 was derived from Quil A, a partially purified mixture of QS saponins containing QS-17, 18, and 21. Similar to QS-21, Quil A enhances a mixed Th1/Th2 immunity with cytotoxic T lymphocyte (CTL) production. Removal of the side chains of all the side chain-containing components in Quil A (e.g., QS-17, QS-18, and QS-21) under basic conditions results in de-acylated product mixture losing its ability to potentiate a Th1 response [72, 82]. However, reintroducing a plain aliphatic side chain (i.e., a dodecylamine side chain), not necessarily in the original acyl side chain position, produces a semisynthetic mixture, GPI-0100, with restored Th1 and CTL capacity [80, 81, 83–85]. GPI-0100 exhibits lower immunostimulatory activity and toxicity in mice compared to Quil A and QS-21, allowing for higher doses than natural QS saponins to achieve the desired immune response without early onset of toxicity. However, GPI-0100’s heterogeneity and variability in content and composition render it unsuitable for clinical use.

**Fig. 3:**
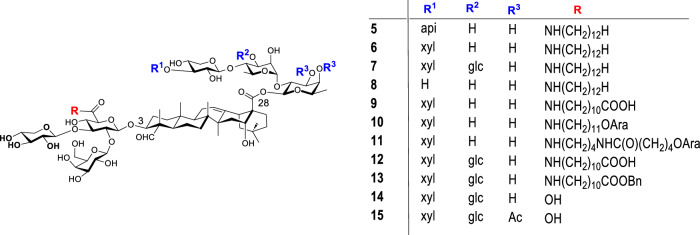
Structure of synthetic QS analogs. api β-D-apiofuranosyl, xyl β-D-xylopyranosyl, glc β-D-glucopyranosyl, Ac acetyl

We synthesized a series of structurally defined QS analogs to conduct SAR studies (Fig. 3). Among them, saponins **5**–**7** corresponded to the GPI-0100 components derived from QS-21_api_, QS-21_xyl_, and QS-17/QS-18, respectively. The adjuvant activities of these synthetic saponins were evaluated in female BALB/c mice, with GPI-0100 serving as a positive control. Groups of mice were immunized with rHagB alone or with an adjuvant via the subcutaneous route (*s.c*.). The recombinant antigen rHagB, a non-fimbrial adhesion hemagglutinin B antigen from the periodontal pathogen *Porphyromonas gingivalis*, is effective in inducing an immune response protective against alveolar bone loss in an experimental animal model [86]. ELISA analysis indicates that synthetic analogs of QS-21, i.e., saponins **5** and **6**, effectively potentiate and maintain a serum IgG response to rHagB following systemic immunization. However, they exhibit weaker activity than GPI-0100 in boosting serum IgG titers, suggesting that GPI-0100’s adjuvant activity may instead be attributed to components derived from other QS saponins such as QS-17 and QS-18 [80, 81].

**Fig. 4:**
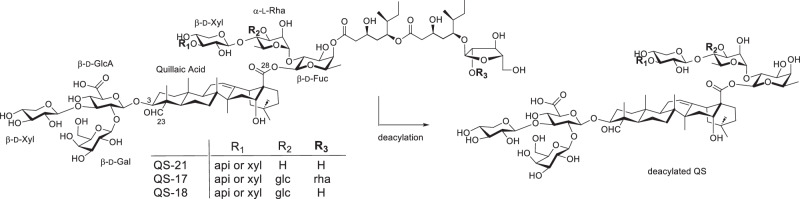
Structure of natural QS saponins and their deacylated counterparts

QS-18 is the most abundant component in the mixture of QS Molina tree bark extract. Its structure differs from QS-21 in the C28 oligosaccharide domain, possessing an extra β-D-glucopyranosyl (glc) unit at the C3 hydroxyl group of the α-L-rhamnopyranosyl (rha) unit (i.e., R^2^ = glc, Fig. 4). On the other hand, QS-17 differs from QS-18 in having a disaccharide unit instead of a monosaccharide unit at the far end of the side chain. QS-17 and QS-18 share the same deacylated residue, thus producing the same analog **7**. Mice immunized with rHagB plus GPI-0100 or rHagB plus analog **7** showed similar IgG1 and IgG2a titers, indicating that QS-17/18 analog **7** is likely the main active component in GPI-0100 [87].

We further investigated the impact of the side chain structure on adjuvant activity. For instance, to enhance water-solubility, the side chains of analogs **9**–**13** are equipped with a polar functional group. ELISA analysis revealed that QS-21 analogs **9**–**11**, and especially **9** and **10**, were effective in potentiating an antigen-specific serum IgG response. The IgG2a/IgG1 ratio of the anti-rHagB responses suggested that rHagB selectively induced a Th2-skewed response following subcutaneous immunization, while GPI-0100, **9**, and **10** potentiated a mixed Th1 and Th2 response to rHagB, and **11** mainly enhanced a Th2 response [88]. Compared with GPI-0100 and analog **7**, both QS-17/18 analogs **12** and **13** with a terminal functionalized side chain induced similar IgG1 titers and relatively lower IgG2a titers [89]. Saponin **14** is deacylated QS-17/18 and serves as the synthetic precursor of **7,** **12**, and **13**. Without a side chain, **14** mainly enhanced IgG1 production; however, by capping the 3-OH and 4-OH of the C28 fucosyl unit with acetyl groups, the newly obtained analog **15** induced similar IgG1 and IgG2a titers to those of QS-21 [90], suggesting another approach to accessing potent QS analogs.

### Discovery of *Momordica* saponin-derived VSA adjuvants

**Fig. 5:**
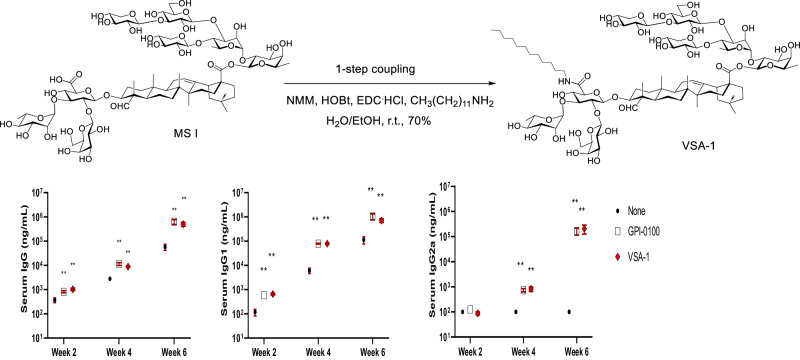
Serum anti-OVA antibody responses in mice immunized with OVA alone or with GPI-0100 (100 μg) or VSA-1 (100 μg) via the *s.c*. route [74]

**Fig. 6:**
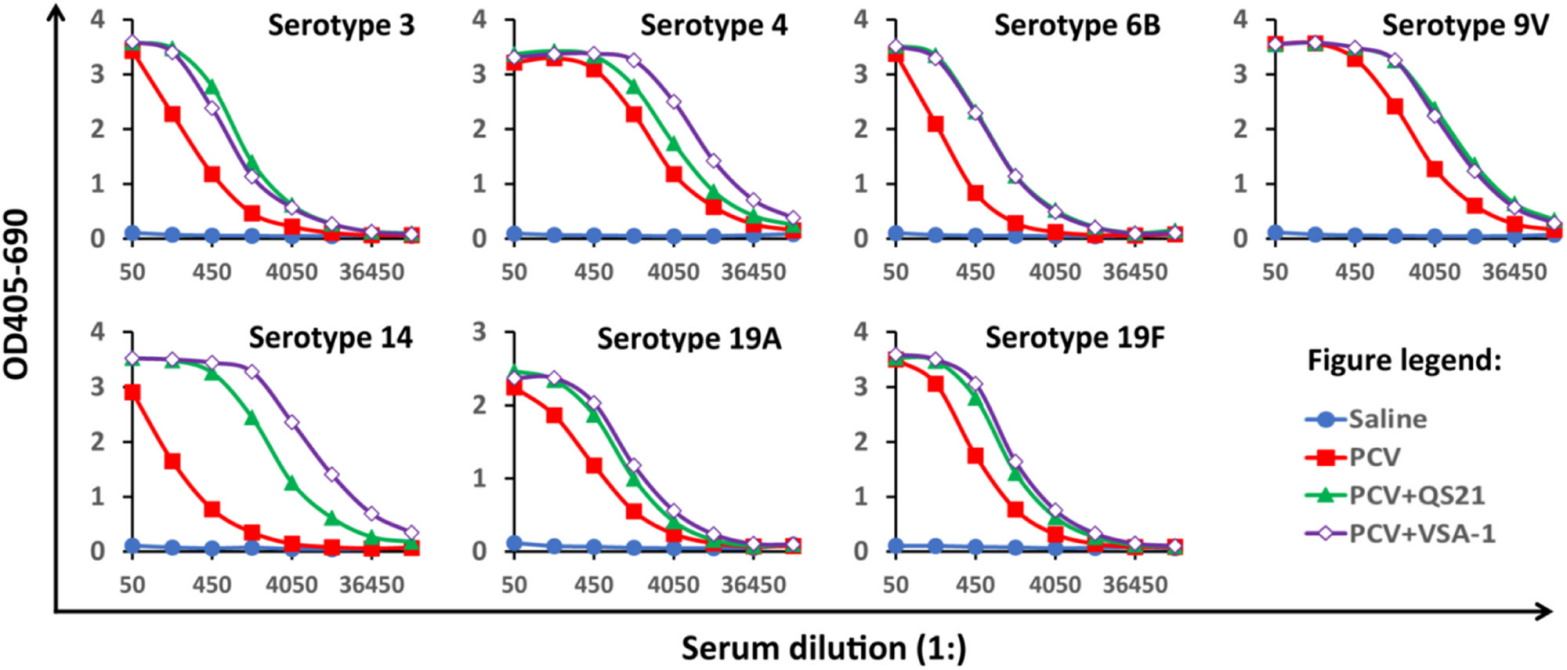
Serum antibody activity to seven serotypes on Day 42. BALB/c mice (8–10 weeks of age, six per group) were immunized via the subcutaneous route (s.c.) on days 0, 14 and 28. Serum samples were collected prior to the first immunization and 2 weeks following the last immunization. The pooled serum samples of each group were analyzed by ELISA

Given the significant difference in adjuvant activity between QS-17/18 and their deacylated counterparts, and the structural similarity between the deacylated QS-17/18 and *Momordica* saponin I (MS I) (Fig. 5) and II (MS II) (Fig. 6) [91], we hypothesized that incorporating a dodecylamine side chain into the natural MS, the corresponding derivatives would result in adjuvant activity resembling that of analog **3**. MS I/II can be obtained from the readily available seeds of *Momordica cochinchinensis* SPRENG (MC), a widely available perennial plant found in regions such as China and Southeast Asia. In traditional Chinese medicine, the seeds, known as *Mubiezi*, are used for the treatment of various conditions including ulcer, mastitis, carbuncle, anal fistula, hemorrhoids, eczema, and neurodermatitis [92].

The composition of the MC seed extract is relatively simple compared to the QS tree bark extract, with a high abundance of MS I/II. This abundance makes isolation more efficient and cost-effective compared to QS saponins. The structures of MS I/II only differ in their triterpenoid cores, with one having a gypsogenin core and the other having a quillaic acid core, similar to immunostimulatory QS-21 and QS-17/18.

MC seed extracts have been investigated for their adjuvant potentials. For instance, a MC seed extract was recently evaluated in a veterinary vaccine candidate against swine foot-and-mouth disease. MC saponins formulated in an oil emulsion were effective in increasing antigen-specific IgG titers in guinea pigs [93]. In another study, MS I/II were compared with other adjuvants in boosting immune responses to a model vaccine containing the F4 fimbriae antigen in chickens. Among the tested adjuvants, Freund’s adjuvant and Quil A were found to be stronger adjuvants than MS I/II in enhancing IgG response in serum and in egg yolk [94]. These results align with observations that the de-acylated QS saponins (e.g., de-acylated QS-17, 18, and 21) with striking structural similarities to MS I/II exhibit reduced adjuvant activities. Therefore, we hypothesize that incorporation of a fatty side chain could potentially transform the weakly active adjuvants MS I/II into potent immunostimulants.

The MS derivatives are synthesized in one step by using a standard amide coupling procedure [95], and the MS I derivative, i.e., VSA-1 (VO_0005326; https://vac.niaid.nih.gov/view?id=41), shows promising adjuvant properties (Fig. 5). In the initial immunological evaluations of VSA-1 as an adjuvant, we used the antigen-specific IgG1 and IgG2a titers as a tentative indication of enhancement of Th2 and Th1 immunity by an adjuvant, given that Th2- or Th1-related cytokines will enhance production of IgG1 or IgG2a, respectively, in BALB/c mice [64]. VSA-1 potentiated antigen-specific IgG1 and IgG2a immune responses that were similar to those induced by GPI-0100, suggesting the similarity between VSA-1 and GPI-0100 in inducing a mixed Th1/Th2 immune response (Fig. 5). Toxicity studies revealed that VSA-1 was significantly less toxic than the natural QS saponins. For example, in an acute toxicity study [95], female BALB/c mice (10 weeks of age) were injected subcutaneously with an adjuvant in 0.1 mL of PBS on the neck. All the mice in the groups treated with VSA-1 (5000 μg) or Quil-A (100 μg or more) died within five days post injection. None of the surviving mice (i.e., with a dose of 2000 μg VSA-1 or less) appeared lethargic by day 7, and no lesion formation was observed. The data indicate that the acute toxicity of VSA-1 is similar to that of GPI-0100 but much lower than that of Quil A.

Recently, we compared VSA-1 and QS-21 in enhancing the clinical glycoconjugate pneumococcal vaccine PCV13 (Prevnar 13 by Pfizer) in mice [96]. *Streptococcus pneumoniae* is a pathogen which causes bacterial pneumonia and meningitis, leading to approximately 660,000/year lower respiratory tract infection-related deaths and 9600/year meningitis-related deaths in adults over 50 years old globally, with high mortality rates in the very young, the elderly, and immunocompromised individuals [97]. Vaccines are deemed effective in preventing infections by *S. pneumoniae*. PCV13 is composed of purified capsular polysaccharides of 13 serotypes of *S. pneumoniae* (*i.e*., serotypes 1, 3, 4, 5, 6A, 6B, 7F, 9V, 14, 19A, 19F, 18C, and 23F) individually conjugated to the diphtheria toxin protein carrier CRM197 [98, 99]. In all age groups, the administration of PCV13 substantially reduced invasive pneumococcal disease (IPD) caused by the serotypes contained within PCV13. However, the effectiveness of IPD reduction varies depending on the serotype and age group. For instance, serotype 3 is associated with the majority of vaccine breakthroughs in children [100–103], while in other cases, serotypes 14 and 19A are responsive for the breakthrough infections [104–107]. Another notable challenge with current pneumococcal vaccines is immunosenescence. Therefore, enhancing the efficacy of PCVs is imperative. To address this, we conducted experiments with groups of BALB/c mice that were subcutaneously immunized with different formulations: saline (negative control), PCV 13 (10% of human dose), PCV13 (10% human dose) combined with QS-21 (20 µg), or PCV 13 (10% of human dose) combined with VSA-1 (100 µg) on days 0, 14 and 28. ELISA analysis demonstrated that PCV13 effectively elicited antigen-specific antibody responses against the seven selected PCV13 serotypes, i.e., 3, 4, 6B, 9V, 14, 19A, and 19F (Fig. 6) [108]. Comparatively, both VSA-1 and QS-21 augmented PCV13-induced antibody responses across these serotypes compared to the PCV13 control group. While their adjuvant activities were similar for serotypes 3, 6B, 9V, 19A, and 19F, VSA-1 outperformed QS-21 for serotypes 4 and 14.

Since opsonic antibodies play a crucial role in PCV-induced immune protection against encapsulated *S. pneumoniae* [109, 110], the opsonophagocytosis assay (OPA) was utilized in this study to assess the capacity of sera to kill the bacteria [108–110]. The OPA data agrees with the ELISA analysis and confirms that the saponin adjuvants are compatible with PCV13 formulation and can effectively improve the vaccine’s efficacy. Both VSA-1 and QS-21 could elevate titers of IgG and OPA for the tested serotypes, including the serotypes that cause PCV13 breakthroughs, such as serotypes 3, 14, and 19A. However, VSA-1 leads to higher opsonic antibody titers for serotypes 4, 6B, 14, and 19A than QS-21 does. Given its advantages over QS-21 (e.g., easy access, sustainable supplies, low toxicity, and high chemical stability), VSA-1 can be a practical immunostimulant to be formulated in a new glycoconjugate pneumococcal vaccine.

Recently, VSA-1, QS-21, and alum were compared for their capacity to enhance the protection induced by an inactivated split influenza virus vaccine in a mouse study [111]. Seasonal influenza viruses infect 5–15% of the global population and contribute to up to 650,000 deaths each year [112]. Although seasonal influenza vaccines can help limit the spread of the influenza virus, there is a high demand for a universal influenza vaccine with effective cross-protection to address challenges posed by continual influenza virus mutation and the threat of another pandemic [113, 114]. A potent immunostimulant is an indispensable vaccine component in achieving this goal. In a recent study, Kang’s laboratory demonstrated that VSA-1 increased the magnitude of virus-specific IgG antibodies and HAI titers induced by an adjuvanted split (sCal) vaccine in C57BL/6 mice after a single dose. Both the VSA-1- or QS-21-adjuvanted vaccines induced higher levels of virus-specific IgG, including HA stalk-specific IgG antibodies, than the alum-adjuvanted vaccine and the vaccine without an adjuvant after prime immunization. However, VSA-1 is more potent than QS-21 in boosting virus-specific IgG1 and IgG2b titers, as well as HAI titers. Specifically, the VSA-1 group exhibited a 2-fold higher HAI titer than the QS-21 group. VSA-1 is similar to QS-21 in boosting IgG2c isotype antibodies. The same study further demonstrated that VSA-1 enhances vaccine-induced protection against homologous influenza virus in mice. Over a period of 14 days after immunization, the VSA-1-adjuvanted group exhibited a 100% survival rate, the least weight loss (approximately 10%), and a rapid recovery to normal weight. Another aspect of vaccine effectiveness assessment is whether it can induce long-lived antibody-secreting cell (ASC) responses. To address this, the study demonstrated that pathogen challenging led to enhanced ASC and IFN-γ-producing T cell responses in the VSA-1-adjuvanted group. Specifically, the VSA-1 group generated more IFN-γ-producing cells than the alum or QS-21 groups.

The study also evaluated the effect of boost immunization with VSA-1-adjuvanted influenza vaccine. The results demonstrated that boost immunization with VSA-1- or QS-21-adjuvanted vaccine produced higher titers of virus-specific IgG, IgG1, IgG2b, and IgG2c antibodies compared to the other groups. The levels of HAI titers at 2 weeks post-boost in mice followed the order of VSA-1 > QS-21 > alum > no adjuvant. Furthermore, VSA-1-adjuvanted boost immunization not only further improved homologous protection but also provided cross-protection against the heterosubtypic rgH5N1 virus.

In the heterosubtypic challenge study, the VSA-1- and QS-21-adjuvanted groups exhibited the highest levels of IgG antibodies after boost, capable of binding the rgH5N1 virus and HA stalk. The VSA-1-adjuvanted group achieved a 100% survival rate, experienced minimal weight loss (approximately 5%), and rapidly recovered to normal weight by day 8 post-challenge. On day 6 after challenge, both the VSA-1 and QS-21 groups displayed virus titers that were 100-fold lower than the naïve infection control group, and approximately 10-fold lower than the un-adjuvanted vaccine group.

Regarding the impact of boost immunization on cellular responses, the data indicated that the VSA-1-adjuvanted Prime-Boost vaccination regimen further enhanced ASC and IFN-γ-producing T cell responses upon virus infection. The regimen induced T cell-dependent protection against heterosubtypic rgH5N1 virus; the VSA-1- and QS-21-adjuvanted groups exhibited higher levels of IFN-γ^+^ and TNF-α^+^-CD4^+^ T and CD8^+^ T cells from lung and spleen tissues compared to the other control groups on day 6 post-challenge with the rgH5N1 virus.

**Fig. 7:**
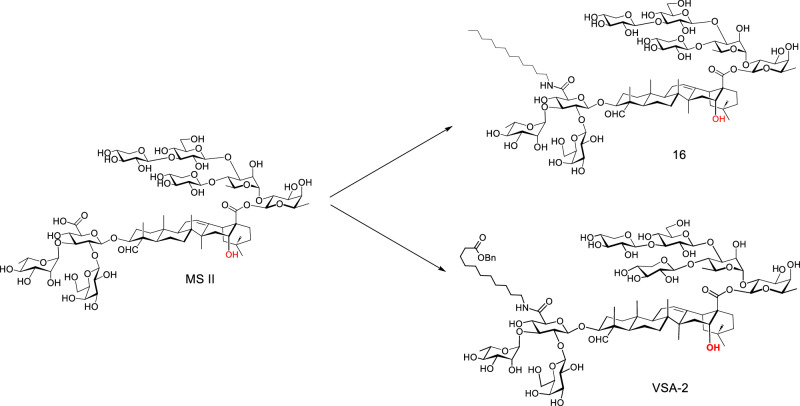
Structure of MS II derivatives **16** and VSA-2

Interestingly, by incorporating the same dodecyl amine side chain in natural saponin MS II, the resulting semisynthetic saponin **16** differs from VSA-1 in adjuvant activity, despite their difference solely at the C16 position of the triterpene core (Fig. 6). Specifically, saponin **16** features a quillaic acid core, while VSA-1 possesses a gypsogenin core. In a head-to-head comparison, VSA-1 and **16** exhibited similar activity in boosting an antigen-specific IgG1 response, but only VSA-1 showed activity in enhancing the IgG2a response. To potentiate the antigen-induced IgG2a response with a MS II-derived semisynthetic saponin, the dodecyl amine side chain was replaced with a new side chain - a 11-aminoundecanoic acid benzyl ester chain - using the same coupling reaction conditions employed for VSA-1 preparation (Fig. 7).

**Fig. 8:**
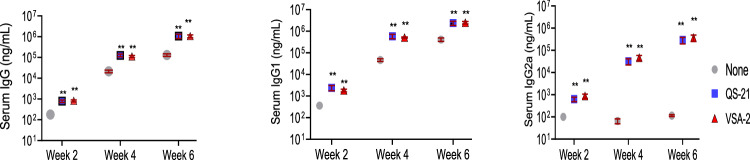
Serum IgG, IgG1, and IgG2a anti-OVA responses in mice immunized by the *s.c*. route with OVA (20.0 µg) alone or with OVA (20.0 µg) in combination with QS-21 (20.0 µg) or VSA-1 (50.0 µg) on days 0, 14 and 28 [96]

The newly obtained saponin adjuvant, VSA-2, was compared with QS-21 in BALB/c mice to assess their capacities for enhancing serum antibody production. The levels of serum antigen-specific antibody activity were determined by ELISA (Fig. 8). These results revealed that VSA-2 and QS-21 similarly potentiated antigen-specific IgG, IgG1, and IgG2a antibody responses [115].

**Fig. 9:**
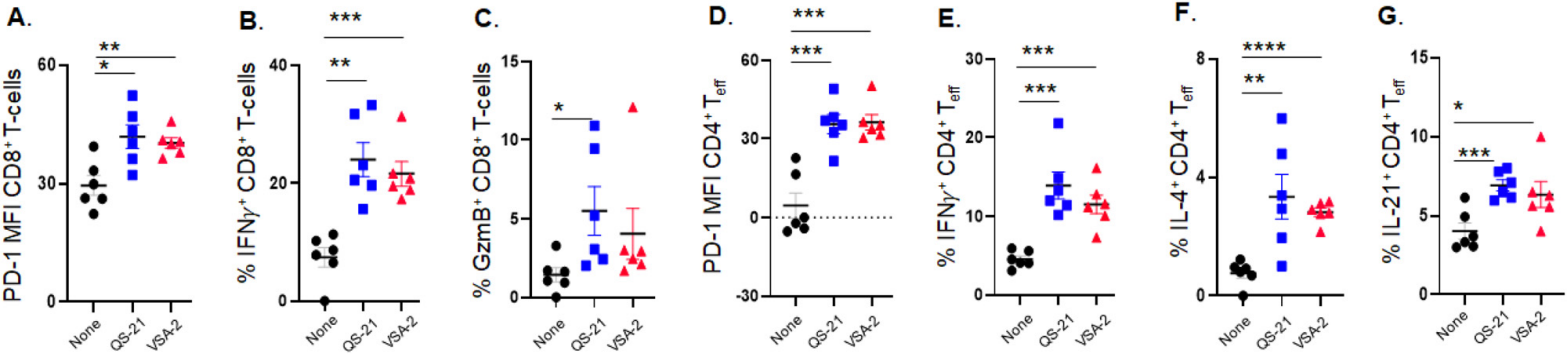
Analysis of mouse spleen cells: CD8^+^ T-cell surface expression of PD-1 (**A**), intracellular levels of IFNγ in CD8^+^ T-cells (**B**), expression of granzyme B in CD8^+^ T-cells (**C**), CD4^+^ T-cell surface expression of PD-1 (**D**), intracellular levels of IFN-γ in CD4^+^ T-cells (**E**), expression of IL-4 in CD4^+^ T-cells (**F**), and expression of IL-21 CD4^+^ T-cells (**G**) [96]

We also collected mouse spleen cells at week 6 post-immunization and analyzed the cellular responses using flow cytometry. VSA-2- and QS-21-adjuvanted groups exhibited higher surface expression of PD-1 (programmed cell death protein 1) and intracellular levels of IFN-γ in both CD8^+^ T cells and CD4^+^ effector T cells (T_eff_). Moreover, these groups showed higher expression of granzyme B in CD8^+^ T cells and higher expression of IL-4 and IL-21 in CD4^+^ T_eff_ cells compared to the antigen-alone group (Fig. 9). The preferential increase in IFN-γ over IL-4 in the VSA-2 and QS-21 groups suggested that the saponin adjuvants induced a Th1-skewed immune response.

**Fig. 10:**
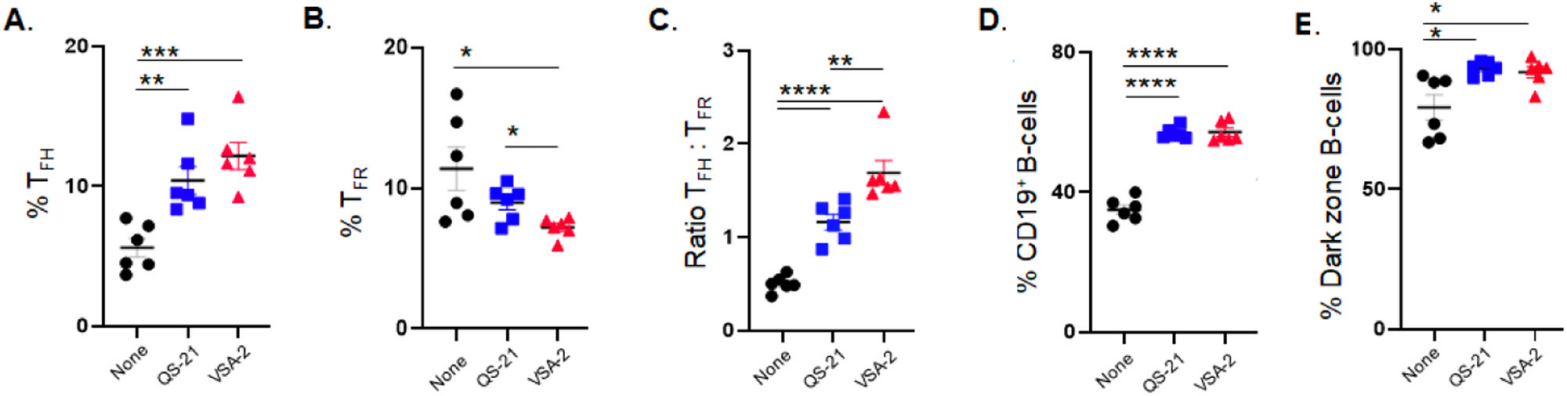
Analysis of mouse spleen cells: T_FH_ cells (**A**), T_FR_ cells (**B**), ratio of T_FH_ to T_RF_ (**C**), CD19^+^ B-cells (**D**), and dark zone B-cells (**E**) [96]

B cells are responsible for antibody production, which is regulated by follicular helper T cells (T_FH_) and follicular regulatory T cells (T_FR_) [116–118]. T_FH_ cells aid germinal center (GC) B-cells in producing high-affinity antibodies, whereas T_FR_ cells suppresses such activity. Analysis of mouse spleen cells revealed that the VSA-2- and QS-21-adjuvanted groups exhibited higher T_FH_ populations (PD-1^+^Bcl6^+^Foxp3^-^CD4^+^CD3^+^) and CD19^+^ B-cell populations (Fig. 10D), along with lower T_FR_ cell populations (PD-1^+^Bcl6^+^Foxp3^+^CD4^+^CD3^+^) (Fig. 10B) compared to the antigen-only group. Correspondingly, the saponin-adjuvanted groups demonstrated higher ratios of T_FH_ cells to T_FR_ cells than the antigen-only control group (Fig. 10C). Additionally, the proportions of dark zone GC B-cells (CXCR4^hi^CD86^lo^Fas^+^GL-7^+^CD19^+^) were higher in both the VSA-2 and QS-21 groups than in the antigen-only group (Fig. 10E). It is recognized that dark zone GC B-cells (CXCR4^hi^CD86^lo^Fas^+^GL-7^+^CD19^+^) are responsible for the producing high-affinity antibodies [119]. These findings suggest that VSA-2 and QS-21 exhibit similar abilities to enhance strong cellular and humoral antibody responses.

## Continuous phytochemical efforts in searching for immunostimulatory natural saponins

Saponins are widely utilized in the food, pharmaceutical, cosmetic industries, and in phytotherapy [120–124]. Alongside medicinal chemistry endeavors to develop a new generation of saponin adjuvants, there have been continuous efforts in phytochemical screenings of natural saponins from various sources to discover more natural saponin immunostimulants. This has led to the discovery of numerous saponins with varying degrees of immunostimulating activities, with some of the immunologically active natural saponins are listed in Table 1. Despite extensive phytochemical efforts, natural saponins with adjuvant properties comparable to QS-21 remain elusive.

**Table 1:** Natural sources of saponins with immunostimulatory activities

Order	Family	Species
Apiales	Araliaceae	*Hedera taurica* [125]*, Panax ginseng* [126–129], *Panax notoginseng* [130, 131]
Asparagales	Asparagaceae	*Asparagus racemosus* [132, 133], *Ophiopogon japonicus* [134], *Asparagus adscendens* [135]
	Iridaceae	*Crocus sativus or saffron* [136]
Asterales	Campanulaceae	*Platycodon grandiflorum* [137–139]
Caryophyllales	Amaranthaceae	*Achyranthes bidentata* [140], *Bupleurum rigidum* [141]*, Chenopodium quinoa* [142]
	Caryophyllaceae	*Acanthophyllum gypsophiloides* [143], *Acanthophyllum squarrosum* [144, 145]*, Allochrusa gypsophiloides* [146], *Gypsophila oldhamiana* [147], *Gypsophila paniculata* [146]*, Saponaria officinalis* [146]*, Silene jenisseensis* [148]
Cucurbitales	Cucurbitaceae	*Bolbostemma paniculatum* [149]*, Gynostemma pentaphyllum* [150]
Dipsacales	Caprifoliaceae	*Cephalaria sumbuliana* [151, 152]
Ericales	Theaceae	*Camellia sinensis* [153]
Fabales	Fabaceae	*Abrus cantoniensis* [154], *Albizia julibrissin* [155], *Astragalus membranaceus or brachycalyx* [156–163], *Calliandra pulcherrima* [164]*, Dolichos Lablab* [165], *Glycine max* [166, 167]*, Glycyrrhiza glabra* [168]*, Glycyrrhiza inflata, and Glycyrrhiza uralensis* [169–171], *Periandra dulcis* [172], *Trigonella* [165]
	Mimosoideae	*Acacia concinna* [173]
	Polygalaceae	*Polygala japonica* [174]*, Polygala senega* [175], *Polygala tenuifolia* [174]
	Quillajaceae	*Quillaja brasiliensis* [176–178]
Ranunculales	Ranunculaceae	*Pulsatilla chinensis* [179]
Sapindales	Sapindaceae	*Aesculus hippocastanum* [165]
Solanales	Solanaceae	*Physalis alkekengi* [180]

## Conclusions

Extensive SAR studies of saponins suggest that incorporating an amide side chain to immunologically inactive or weakly active saponins is a viable approach for generating robust immunostimulants. Natural bisdesmosidic saponin precursors, such as *Momordica* saponins, can be conveniently obtained from sustainable sources. This strategy not only helps address the supply shortage of QS-21 but also provides an opportunity to access a broad range of structurally defined and homogenous saponin adjuvants. These adjuvants can complement QS-21 with different adjuvant properties to meet the requirements of various vaccine applications. Furthermore, semisynthetic saponin adjuvants offer new opportunities for developing molecular probes crucial for the exploration of the molecular mechanism underlying saponins’ adjuvanticity. They also facilitate the development of various chemical combinations of adjuvants and built-in adjuvants, thereby enabling the creation of multicomponent synthetic vaccines.
